# 2-Hydroxy-*N*,*N*′-diisopropylpropane-1,3-diaminium dichloride

**DOI:** 10.1107/S1600536810023585

**Published:** 2010-06-23

**Authors:** Xuehui Hou, Likui Zhao

**Affiliations:** a Department of Quality Detection and Management, Zhengzhou College of Animal Husbandry Engineering, Zhengzhou 450011, People’s Republic of China

## Abstract

In the crystal structure of the title amino alcohol derivative, C_9_H_24_N_2_O^2+^·2Cl^−^, the cations and anions are linked by inter­molecular O—H⋯Cl and N—H⋯Cl hydrogen bonds into a three-dimensional network.

## Related literature

For the applications of amino alcohols and their derivatives in organic synthesis, see: Ellison & Gandhi (2005 ▶); Li *et al.* (2004 ▶). 
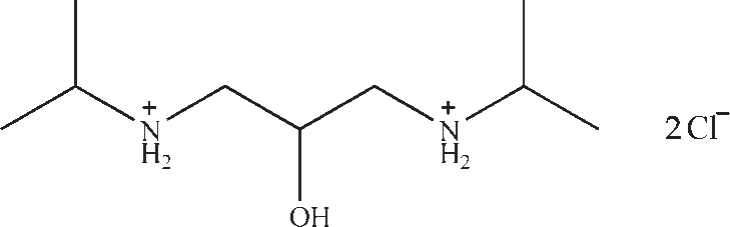


## Experimental

### 

#### Crystal data

C_9_H_24_N_2_O^2+^·2Cl^−^
*M* *_r_* = 247.20
Triclinic, 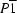

*a* = 6.240 (1) Å
*b* = 10.0081 (14) Å
*c* = 11.3519 (16) Å
α = 86.198 (1)°
β = 88.052 (2)°
γ = 83.308 (1)°
*V* = 702.31 (18) Å^3^
*Z* = 2
Mo *K*α radiation
μ = 0.44 mm^−1^
*T* = 298 K
0.50 × 0.45 × 0.44 mm

#### Data collection

Siemens SMART CCD area-detector diffractometer
Absorption correction: multi-scan (*SADABS*; Sheldrick, 1996 ▶) *T*_min_ = 0.810, *T*_max_ = 0.830
3684 measured reflections
2448 independent reflections
1999 reflections with *I* > 2σ(*I*)
*R*_int_ = 0.016

#### Refinement

*R*[*F* ^2^ > 2σ(*F* ^2^)] = 0.034
*wR*(*F* ^2^) = 0.096
*S* = 1.03
2448 reflections
131 parameters
H-atom parameters constrained
Δρ_max_ = 0.25 e Å^−3^
Δρ_min_ = −0.23 e Å^−3^

### 

Data collection: *SMART* (Siemens, 1996 ▶); cell refinement: *SAINT* (Siemens, 1996 ▶); data reduction: *SAINT*; program(s) used to solve structure: *SHELXS97* (Sheldrick, 2008 ▶); program(s) used to refine structure: *SHELXL97* (Sheldrick, 2008 ▶); molecular graphics: *SHELXTL* (Sheldrick, 2008 ▶); software used to prepare material for publication: *SHELXTL*.

## Supplementary Material

Crystal structure: contains datablocks I, global. DOI: 10.1107/S1600536810023585/rz2467sup1.cif

Structure factors: contains datablocks I. DOI: 10.1107/S1600536810023585/rz2467Isup2.hkl

Additional supplementary materials:  crystallographic information; 3D view; checkCIF report

## Figures and Tables

**Table 1:** Hydrogen-bond geometry (Å, °)

*D*—H⋯*A*	*D*—H	H⋯*A*	*D*⋯*A*	*D*—H⋯*A*
O1—H1⋯Cl1^i^	0.82	2.33	3.1445 (15)	172
N2—H2*B*⋯Cl1^i^	0.90	2.24	3.1336 (15)	173
N2—H2*A*⋯Cl2^ii^	0.90	2.22	3.1130 (15)	172
N1—H1*B*⋯Cl2^iii^	0.90	2.20	3.0920 (15)	174
N1—H1*A*⋯Cl1^iv^	0.90	2.38	3.2119 (16)	154

Symmetry codes: (i) 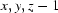; (ii) 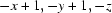; (iii) 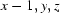; (iv) 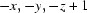.

**Fig. 1.**
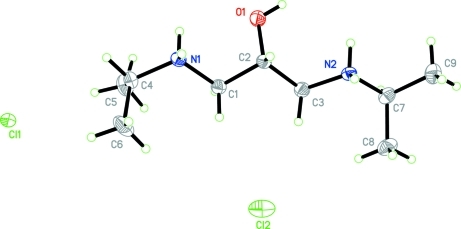
The molecular structure of the compound, with atom labels and 50% probability displacement ellipsoids.

**Fig. 2.**
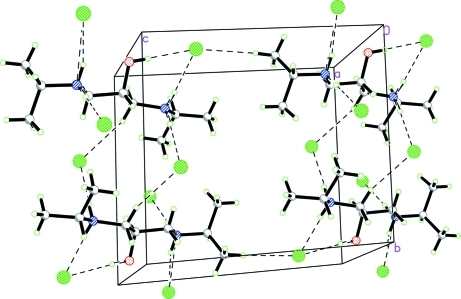
Crystal packing of the title compound. showing a three-dimensional structure linked by hydrogen bonds (dashed lines).

## supplementary crystallographic information

### Comment

Amino alcohols are important structural elements for the asymmetric catalysis of chiral ligands (Li *et al.*, 2004) as well as of biologically active compounds (Ellison & Gandhi, 2005). In order to develop new applications for amino alcohols and their derivatives, structural modifications of these compounds have been extensively investigated. As a contribution in this filed, we report here the crystal structure of the title compound.

The molecular structure of the title compound is shown in Fig. 1. Bond lengths and angles in the cation are not unusual. In the crystal packing (Fig. 2), intermolecular O—H···Cl and N—H···Cl hydrogen bonds (Table 1) link molecules into a three-dimensional network.

### Experimental

To a solution of isopropamide (24.0 g, 0.4 mol) in acetone (200 ml), epichlorohydrin (9.2 g, 0.1 mol) and K_2_CO_3_ (13.8 g, 0.1 mol) were added. The mixture was stirred at room temperature for 8 h, followed by filtration and purification by crystallization from ethyl acetate, giving title compound as colourless single crystals suitable for X-ray analysis.

### Refinement

All H atoms were placed geometrically and treated as riding on their parent atoms, with C—H = 0.93–0.98 Å, N—H = 0.90 Å, O—H = 0.82 Å, and with *U*_iso_(H) = 1.2 *U*_eq_(C, N) or 1.5 *U*_eq_(C, O) for methyl and hydroxy H atoms.

### Crystal data

**Table:**

C_9_H_24_N_2_O^2+^·2Cl^−^	*Z* = 2
*M**_r_* = 247.20	*F*(000) = 268
Triclinic, *P*1	*D*_x_ = 1.169 Mg m^−^^3^
Hall symbol: -P 1	Mo *K*α radiation, λ = 0.71073 Å
*a* = 6.240 (1) Å	Cell parameters from 2066 reflections
*b* = 10.0081 (14) Å	θ = 2.6–27.6°
*c* = 11.3519 (16) Å	µ = 0.44 mm^−^^1^
α = 86.198 (1)°	*T* = 298 K
β = 88.052 (2)°	Block, colourless
γ = 83.308 (1)°	0.50 × 0.45 × 0.44 mm
*V* = 702.31 (18) Å^3^	

### Data collection

**Table:**

Siemens SMART CCD area-detector diffractometer	2448 independent reflections
Radiation source: fine-focus sealed tube	1999 reflections with *I* > 2σ(*I*)
graphite	*R*_int_ = 0.016
phi and ω scans	θ_max_ = 25.0°, θ_min_ = 1.8°
Absorption correction: multi-scan (*SADABS*; Sheldrick, 1996)	*h* = −7→5
*T*_min_ = 0.810, *T*_max_ = 0.830	*k* = −11→11
3684 measured reflections	*l* = −13→10

### Refinement

**Table:**

Refinement on *F*^2^	Primary atom site location: structure-invariant direct methods
Least-squares matrix: full	Secondary atom site location: difference Fourier map
*R*[*F*^2^ > 2σ(*F*^2^)] = 0.034	Hydrogen site location: inferred from neighbouring sites
*wR*(*F*^2^) = 0.096	H-atom parameters constrained
*S* = 1.03	*w* = 1/[σ^2^(*F*_o_^2^) + (0.048*P*)^2^ + 0.1942*P*] where *P* = (*F*_o_^2^ + 2*F*_c_^2^)/3
2448 reflections	(Δ/σ)_max_ < 0.001
131 parameters	Δρ_max_ = 0.25 e Å^−^^3^
0 restraints	Δρ_min_ = −0.23 e Å^−^^3^

### Special details

**Table:**

Geometry. All e.s.d.'s (except the e.s.d. in the dihedral angle between two l.s. planes) are estimated using the full covariance matrix. The cell e.s.d.'s are taken into account individually in the estimation of e.s.d.'s in distances, angles and torsion angles; correlations between e.s.d.'s in cell parameters are only used when they are defined by crystal symmetry. An approximate (isotropic) treatment of cell e.s.d.'s is used for estimating e.s.d.'s involving l.s. planes.
Refinement. Refinement of *F*^2^ against ALL reflections. The weighted *R*-factor *wR* and goodness of fit *S* are based on *F*^2^, conventional *R*-factors *R* are based on *F*, with *F* set to zero for negative *F*^2^. The threshold expression of *F*^2^ > σ(*F*^2^) is used only for calculating *R*-factors(gt) *etc*. and is not relevant to the choice of reflections for refinement. *R*-factors based on *F*^2^ are statistically about twice as large as those based on *F*, and *R*- factors based on ALL data will be even larger.

### Fractional atomic coordinates and isotropic or equivalent isotropic displacement parameters (Å^2^)

**Table:**

	*x*	*y*	*z*	*U*_iso_*/*U*_eq_	
Cl1	0.19859 (8)	0.04318 (5)	0.75891 (4)	0.04461 (17)	
Cl2	0.73770 (9)	0.43851 (5)	0.17643 (6)	0.0585 (2)	
N1	0.0878 (2)	0.20330 (14)	0.24284 (12)	0.0307 (3)	
H1A	0.0376	0.1276	0.2223	0.037*	
H1B	−0.0074	0.2733	0.2183	0.037*	
N2	0.4576 (2)	0.26100 (14)	−0.14466 (12)	0.0302 (3)	
H2A	0.3877	0.3447	−0.1542	0.036*	
H2B	0.3742	0.2041	−0.1738	0.036*	
O1	0.1777 (3)	0.09416 (15)	0.02964 (12)	0.0524 (4)	
H1	0.1697	0.0850	−0.0413	0.079*	
C1	0.2960 (3)	0.21572 (19)	0.17837 (16)	0.0345 (4)	
H1C	0.4012	0.1409	0.2033	0.041*	
H1D	0.3503	0.2987	0.1968	0.041*	
C2	0.2656 (3)	0.21601 (17)	0.04656 (15)	0.0316 (4)	
H2	0.1629	0.2932	0.0209	0.038*	
C3	0.4813 (3)	0.22602 (19)	−0.01638 (16)	0.0355 (4)	
H3A	0.5534	0.2942	0.0182	0.043*	
H3B	0.5710	0.1405	−0.0051	0.043*	
C4	0.0935 (3)	0.1991 (2)	0.37596 (16)	0.0394 (5)	
H4	−0.0526	0.1880	0.4065	0.047*	
C5	0.2409 (4)	0.0782 (2)	0.4228 (2)	0.0577 (6)	
H5A	0.2060	−0.0012	0.3880	0.087*	
H5B	0.2223	0.0675	0.5071	0.087*	
H5C	0.3881	0.0913	0.4031	0.087*	
C6	0.1505 (5)	0.3315 (2)	0.4160 (2)	0.0646 (7)	
H6A	0.2959	0.3434	0.3908	0.097*	
H6B	0.1385	0.3309	0.5006	0.097*	
H6C	0.0534	0.4043	0.3821	0.097*	
C7	0.6661 (3)	0.25586 (19)	−0.21644 (18)	0.0385 (4)	
H7	0.7421	0.1646	−0.2069	0.046*	
C8	0.8085 (3)	0.3540 (2)	−0.1736 (2)	0.0548 (6)	
H8A	0.7330	0.4433	−0.1790	0.082*	
H8B	0.9383	0.3518	−0.2216	0.082*	
H8C	0.8445	0.3291	−0.0929	0.082*	
C9	0.6110 (4)	0.2857 (3)	−0.34475 (19)	0.0578 (6)	
H9A	0.5186	0.2223	−0.3678	0.087*	
H9B	0.7412	0.2783	−0.3926	0.087*	
H9C	0.5383	0.3754	−0.3554	0.087*	

### Atomic displacement parameters (Å^2^)

**Table:**

	*U*^11^	*U*^22^	*U*^33^	*U*^12^	*U*^13^	*U*^23^
Cl1	0.0475 (3)	0.0492 (3)	0.0417 (3)	−0.0200 (2)	0.0034 (2)	−0.0139 (2)
Cl2	0.0460 (3)	0.0349 (3)	0.0946 (5)	0.0039 (2)	−0.0206 (3)	−0.0090 (3)
N1	0.0315 (8)	0.0292 (8)	0.0316 (8)	−0.0018 (6)	−0.0026 (6)	−0.0046 (6)
N2	0.0298 (8)	0.0272 (7)	0.0345 (8)	−0.0060 (6)	−0.0002 (6)	−0.0043 (6)
O1	0.0714 (10)	0.0555 (9)	0.0375 (8)	−0.0341 (8)	0.0079 (7)	−0.0129 (7)
C1	0.0317 (10)	0.0387 (10)	0.0336 (10)	−0.0049 (8)	−0.0013 (8)	−0.0051 (8)
C2	0.0328 (10)	0.0309 (9)	0.0319 (10)	−0.0050 (7)	−0.0029 (8)	−0.0031 (7)
C3	0.0337 (10)	0.0371 (10)	0.0358 (10)	−0.0044 (8)	−0.0030 (8)	−0.0010 (8)
C4	0.0432 (11)	0.0474 (11)	0.0284 (10)	−0.0070 (9)	0.0023 (8)	−0.0065 (8)
C5	0.0689 (16)	0.0640 (15)	0.0370 (12)	0.0020 (12)	−0.0064 (11)	0.0072 (10)
C6	0.0913 (19)	0.0615 (15)	0.0450 (13)	−0.0129 (13)	−0.0062 (13)	−0.0226 (11)
C7	0.0317 (10)	0.0351 (10)	0.0479 (12)	−0.0020 (8)	0.0083 (8)	−0.0046 (9)
C8	0.0344 (11)	0.0639 (15)	0.0677 (15)	−0.0160 (10)	0.0017 (10)	0.0003 (12)
C9	0.0636 (15)	0.0685 (15)	0.0427 (13)	−0.0159 (12)	0.0136 (11)	−0.0068 (11)

### Geometric parameters (Å, °)

**Table:**

N1—C1	1.483 (2)	C4—C6	1.513 (3)
N1—C4	1.510 (2)	C4—H4	0.9800
N1—H1A	0.9000	C5—H5A	0.9600
N1—H1B	0.9000	C5—H5B	0.9600
N2—C3	1.483 (2)	C5—H5C	0.9600
N2—C7	1.509 (2)	C6—H6A	0.9600
N2—H2A	0.9000	C6—H6B	0.9600
N2—H2B	0.9000	C6—H6C	0.9600
O1—C2	1.421 (2)	C7—C9	1.510 (3)
O1—H1	0.8200	C7—C8	1.512 (3)
C1—C2	1.514 (2)	C7—H7	0.9800
C1—H1C	0.9700	C8—H8A	0.9600
C1—H1D	0.9700	C8—H8B	0.9600
C2—C3	1.513 (2)	C8—H8C	0.9600
C2—H2	0.9800	C9—H9A	0.9600
C3—H3A	0.9700	C9—H9B	0.9600
C3—H3B	0.9700	C9—H9C	0.9600
C4—C5	1.511 (3)		
			
C1—N1—C4	116.24 (14)	N1—C4—H4	107.3
C1—N1—H1A	108.2	C5—C4—H4	107.3
C4—N1—H1A	108.2	C6—C4—H4	107.3
C1—N1—H1B	108.2	C4—C5—H5A	109.5
C4—N1—H1B	108.2	C4—C5—H5B	109.5
H1A—N1—H1B	107.4	H5A—C5—H5B	109.5
C3—N2—C7	115.27 (14)	C4—C5—H5C	109.5
C3—N2—H2A	108.5	H5A—C5—H5C	109.5
C7—N2—H2A	108.5	H5B—C5—H5C	109.5
C3—N2—H2B	108.5	C4—C6—H6A	109.5
C7—N2—H2B	108.5	C4—C6—H6B	109.5
H2A—N2—H2B	107.5	H6A—C6—H6B	109.5
C2—O1—H1	109.5	C4—C6—H6C	109.5
N1—C1—C2	110.12 (14)	H6A—C6—H6C	109.5
N1—C1—H1C	109.6	H6B—C6—H6C	109.5
C2—C1—H1C	109.6	N2—C7—C9	108.02 (16)
N1—C1—H1D	109.6	N2—C7—C8	110.37 (16)
C2—C1—H1D	109.6	C9—C7—C8	112.24 (18)
H1C—C1—H1D	108.1	N2—C7—H7	108.7
O1—C2—C3	113.58 (15)	C9—C7—H7	108.7
O1—C2—C1	105.22 (14)	C8—C7—H7	108.7
C3—C2—C1	108.81 (14)	C7—C8—H8A	109.5
O1—C2—H2	109.7	C7—C8—H8B	109.5
C3—C2—H2	109.7	H8A—C8—H8B	109.5
C1—C2—H2	109.7	C7—C8—H8C	109.5
N2—C3—C2	112.00 (14)	H8A—C8—H8C	109.5
N2—C3—H3A	109.2	H8B—C8—H8C	109.5
C2—C3—H3A	109.2	C7—C9—H9A	109.5
N2—C3—H3B	109.2	C7—C9—H9B	109.5
C2—C3—H3B	109.2	H9A—C9—H9B	109.5
H3A—C3—H3B	107.9	C7—C9—H9C	109.5
N1—C4—C5	110.60 (16)	H9A—C9—H9C	109.5
N1—C4—C6	110.44 (16)	H9B—C9—H9C	109.5
C5—C4—C6	113.51 (19)		
			
C4—N1—C1—C2	178.96 (14)	C1—C2—C3—N2	−165.75 (14)
N1—C1—C2—O1	−56.87 (18)	C1—N1—C4—C5	−61.5 (2)
N1—C1—C2—C3	−178.91 (14)	C1—N1—C4—C6	65.0 (2)
C7—N2—C3—C2	−172.65 (14)	C3—N2—C7—C9	176.59 (16)
O1—C2—C3—N2	77.43 (19)	C3—N2—C7—C8	−60.4 (2)

### Hydrogen-bond geometry (Å, °)

**Table:**

*D*—H···*A*	*D*—H	H···*A*	*D*···*A*	*D*—H···*A*
O1—H1···Cl1^i^	0.82	2.33	3.1445 (15)	172
N2—H2B···Cl1^i^	0.90	2.24	3.1336 (15)	173
N2—H2A···Cl2^ii^	0.90	2.22	3.1130 (15)	172
N1—H1B···Cl2^iii^	0.90	2.20	3.0920 (15)	174
N1—H1A···Cl1^iv^	0.90	2.38	3.2119 (16)	154

Symmetry codes: (i) *x*, *y*, *z*−1; (ii) −*x*+1, −*y*+1, −*z*; (iii) *x*−1, *y*, *z*; (iv) −*x*, −*y*, −*z*+1.

## References

[bb1] Ellison, K. E. & Gandhi, G. (2005). *Drugs*, pp. 787–797. 10.2165/00003495-200565060-00006 15819591

[bb2] Li, Y., He, B., Qin, B., Feng, X. M. & Zhang, G. L. (2004). *J. Org. Chem.* **69**, 7910–7913. 10.1021/jo0488356 15527269

[bb3] Sheldrick, G. M. (1996). *SADABS* University of Göttingen, Germany.

[bb4] Sheldrick, G. M. (2008). *Acta Cryst.* A**64**, 112–122. 10.1107/S0108767307043930 18156677

[bb5] Siemens (1996). *SMART* and *SAINT* Siemens Analytical X-ray Systems Inc., Madison, Wisconsin, USA.

